# High prevalence of genotype G in HIV co-infected patients compared with HBV monoinfected patients in México

**DOI:** 10.1186/1758-2652-13-S4-P201

**Published:** 2010-11-08

**Authors:** JA Mata-Marín, JE Gaytán-Martínez, CI Arroyo-Anduiza, G Calderón-Rodriguez

**Affiliations:** 1 Hospital de Infectología "La Raza" National Medical Center/IMSS, Infectious diseases, Mexico City, Mexico; 2 Hospital de Cardiología/IMSS, Clinical Pathology, Mexico City, Mexico; 3 UIMII "La Raza" National Medical Center/IMSS, Seris y Jacarandas s/n, Col La Raza, Mexico City, Mexico

## Background

It has been reported in Mexico that genotypes H and G are the most common, although genotypes prevalence in HIV co-infected patients is unknown.

## Purpose of the study

We estimated the prevalence and identified the resistance pattern of HBV/H and G genotypes in HIV co-infected patients and compared them in mono-infected HBV patients.

## Methods

A cross-sectional prevalence and analytic study were realized. Risk factors, HIV or HCV co-infections, antiretroviral therapy (ART) experience, HBsAg, HBeAg, HBV viral load and mutations genetic analysis were collected; CD4+ cells count from HIV co-infected patients and HIV viral load were measured. We calculated the prevalence and exact 95% binomial confidence interval as well the Odds ratios (OR) and 95% confidence intervals to assess the relationship between risks factors and the risk of having HBV/H or G genotype.

## Results

We enrolled 84 patients, 72 men and 12 women with 41 HIV co-infected patients. The distribution of HBV genotypes was: HBV/H 56 (66%), HBV/G 22 (26.1% [95% CI 17% to 36%]), HBV/F 4 (4.7%) and HBV/A 2 (2.3%). The most frequent mutations presented in 9 HIV co-infected patients and one mono-infected patient with ART experience were rtM204V and seven of them showed genotype G (7/9). Mono-infected HBV patients exposed more probability to HBV/H genotype than co-infected HIV patients OR 13.0 (CI 95% 3.40-49.79), P=0.0001 In contrast co-infected patients presented less possibility to have genotype H, 0.56 (CI 95% 0.42-0.75).

## Conclusions

Our results suggest that HBV/G genotype predominates in co-infected patients. As well, rtM204V and rtL180M mutations are common in HBV/HIV co-infected patients with genotype G and ART experience.

